# Mito-nuclear co-evolution: the positive and negative sides of functional ancient mutations

**DOI:** 10.3389/fgene.2014.00448

**Published:** 2014-12-23

**Authors:** Liron Levin, Amit Blumberg, Gilad Barshad, Dan Mishmar

**Affiliations:** Department of Life Sciences, Ben-Gurion University of the NegevBeersheba, Israel

**Keywords:** mito-nuclear co-evolution, mtDNA, nuclear DNA, RNM, SNM

## Abstract

Most cell functions are carried out by interacting factors, thus underlying the functional importance of genetic interactions between genes, termed epistasis. Epistasis could be under strong selective pressures especially in conditions where the mutation rate of one of the interacting partners notably differs from the other. Accordingly, the order of magnitude higher mitochondrial DNA (mtDNA) mutation rate as compared to the nuclear DNA (nDNA) of all tested animals, should influence systems involving mitochondrial-nuclear (mito-nuclear) interactions. Such is the case of the energy producing oxidative phosphorylation (OXPHOS) and mitochondrial translational machineries which are comprised of factors encoded by both the mtDNA and the nDNA. Additionally, the mitochondrial RNA transcription and mtDNA replication systems are operated by nDNA-encoded proteins that bind mtDNA regulatory elements. As these systems are central to cell life there is strong selection toward mito-nuclear co-evolution to maintain their function. However, it is unclear whether (A) mito-nuclear co-evolution befalls only to retain mitochondrial functions during evolution or, also, (B) serves as an adaptive tool to adjust for the evolving energetic demands as species’ complexity increases. As the first step to answer these questions we discuss evidence of both negative and adaptive (positive) selection acting on the mtDNA and nDNA-encoded genes and the effect of both types of selection on mito-nuclear interacting factors. Emphasis is given to the crucial role of recurrent ancient (nodal) mutations in such selective events. We apply this point-of-view to the three available types of mito-nuclear co-evolution: protein–protein (within the OXPHOS system), protein-RNA (mainly within the mitochondrial ribosome), and protein-DNA (at the mitochondrial replication and transcription machineries).

## INTRODUCTION

Disease-causing mutations are, in general, negatively selected. As a result such mutations reoccur on different genetic backgrounds, as they cannot become prevalent, unless they cause recessive disorders and could survive in a heterozygous state. Hence, dominant disorders, which lead to phenotypes in a heterozygous state, will be subjected to stronger negative selection. Accordingly, evolutionary survival of a dominant deleterious mutation in two related lineages depends on the penetrance and severity of the phenotype. That is, unless the functionality of such mutations is compensated either by additional genetic changes within the same gene or by genetically interacting (epistatic) factors (Azevedo et al., 2006).

Since their first discovery, disease-causing mutations in the maternally inherited mitochondrial genome (mtDNA) indeed cause severe phenotypes, but their phenotypic penetrance is notably partial (Holt et al., 1988; Wallace et al., 1988a,b), suggesting the involvement of epistatic modifiers. Because of uni-parental inheritance, mtDNA mutations cannot be transmitted at a heterozygous state, and most have to re-occur in order to be identified in unrelated families. However, during the past decade, several human disease-causing mutations both in the mtDNA and in the nuclear genome (nDNA) were identified as common polymorphisms, which define phylogenetic nodes, in other species (Kern and Kondrashov, 2004; de Magalhaes, 2005; Azevedo et al., 2006, 2009). It has been suggested that this phenomenon could be explained by the pre-occurrence of compensatory mutations either within the same gene or in epistatically interacting genes. Candidates for such compensations have been identified in the species harboring human disease-causing mutations as polymorphisms (Azevedo et al., 2009). If this is the case, then one could envision that a similar mechanism could be applied to the survival of common polymorphisms in humans having functional properties comparable to disease-causing mutations. Indeed, we identified such mutations in the human mtDNA (Levin et al., 2013). In order to consider the mechanisms that enabled their long-time survival, one should be better acquainted with the mitochondrial genetic system.

### MITOCHONDRIAL GENETICS – OVERVIEW

The mitochondrion is the major source of cellular energy (Wallace, 2007). The vast majority of eukaryotic cells cannot survive without the mitochondria and the mitochondrion cannot survive independently of its host cell. The mitochondrion is the only organelle in animal cells with its own genome and is believed to have originated from alpha-proteobacteria (Lane and Martin, 2010; Gray, 2012). However, mtDNA of higher eukaryotes harbors only a subset of the genes essential for mitochondrial activity. Hence, vertebrate mtDNA harbors just 37 genes (see below), while the remaining genes (*N* = ∼1500) required for mitochondrial function (such as apoptosis, nucleotide biosynthesis, fatty acids metabolism, the metabolism of iron etc.) are encoded by the nDNA (Calvo and Mootha, 2010). These nDNA genes are translated in the cytoplasm and, in turn, imported into the mitochondrion via import machineries.

What is the origin of these mitochondrial nDNA-encoded genes? It is possible that during the course of evolution many previously existing nDNA genes acquired their mitochondrial functions subsequent to the incorporation of the mitochondrion. A non-opposing alternative is that genes with mitochondrial functions were once encoded by the bacterial ancestor, and were gradually relocated to the host nucleus during the course of time. Approximately 2 billion years have passed since the occurrence of the symbiotic event that gave rise to all Eukaryotes (Gray, 2012) lending plenty of time for both processes to have occurred in parallel.

### MITOCHONDRIAL GENETICS AND GENOMICS

**FIGURE 1:**
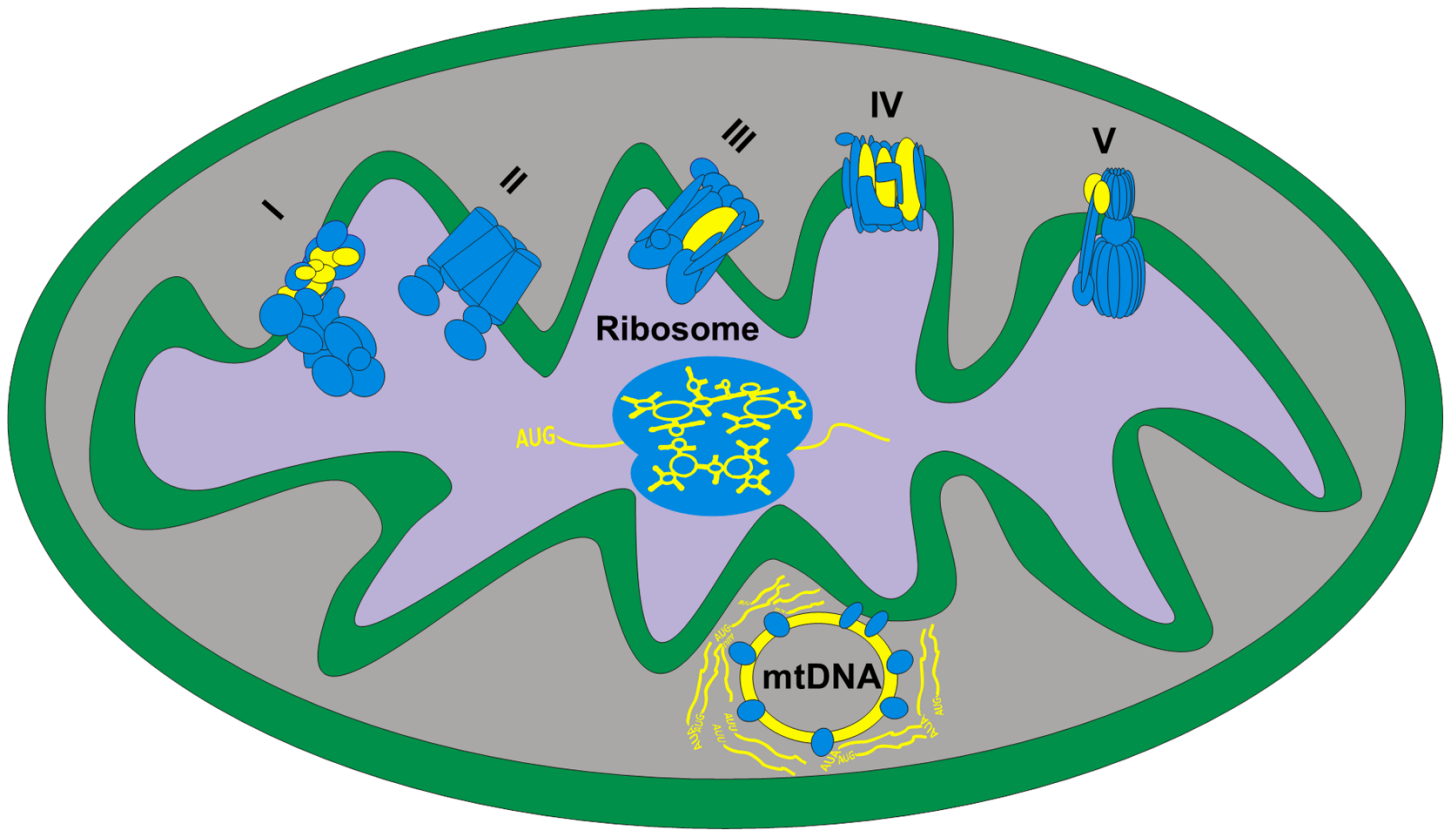
**Human mitochondrial systems require cooperation of nDNA and mtDNA-encoded factors – an illustration of a single mitochondrion.** Shown are the five OXPHOS complexes, the mitochondrial ribosome and the mtDNA with its adjacent proteins and RNA products, representing the replication and transcription systems. Blue objects – nDNA-encoded proteins that are imported into the mitochondria. Yellow objects – mtDNA-encoded elements including the mtDNA itself, mitochondrial RNAs and the mtDNA-encoded OXPHOS complexes’ subunits. I–V: OXPHOS protein complexes. The physical location of the mtDNA and ‘cloud’ of synthesized mRNA is based on our interpretation of recent high resolution microscopy investigation of the mitochondrial nucleoid, i.e., the mtDNA-protein structure that encompasses the replication and transcription machineries (Brown et al., 2011).

Although most of the functions required for the various activities of the mitochondria are encoded solely by nDNA genes, two mitochondrial machineries are comprised of both mtDNA and nDNA-encoded genes: the oxidative phosphorylation ATP production system (OXPHOS) and the mitochondrial-specific protein translational machinery (**Figure 1**). The small, circular mtDNA encodes for 37 genes in vertebrates: (A) 13 genes coding for polypeptide members of four out of the five multi-subunit OXPHOS protein complexes. These include seven protein subunits (*ND1-6*, *ND4L*) of NADH ubiquinone oxidoreductase (OXPHOS complex I), one subunit (*CytB*) of cytochrome bc1 (OXPHOS complex III), three subunits (*COI-III*) of cytochrome c oxidase (OXPHOS complex IV) and two subunits (*ATP6,8*) of F1-F0 ATP synthase (OXPHOS complex V); (B) Two rRNA genes (*12SrRNA* and 1*6SrRNA*), which are constituents of the mitochondrial ribosome; and (C) 22 tRNA genes. These 37 factors encompass nearly ∼93% of the vertebrates’ mtDNA, although species may vary in gene order (Wallace, 2007). The rest of the mtDNA (∼7%) comprises non-coding sequences which harbor regulatory elements. The D-Loop (∼1000 bp in length), which is the larger non-coding region, includes the heavy and light mtDNA strands promoters (HSP and LSP, respectively) as well as the origin of replication for the heavy strand (Ori-H). Another shorter non-coding region encompasses the light strand origin of replication (Ori-L), which is located ∼5000 base pairs apart from the D-loop. Although the HSP and LSP are separated in most vertebrates, the mtDNAs of most birds and the African clawed frog (*Xenopus laevis*) have a bidirectional promoter that controls the transcription of both mtDNA strands. Such organization raises the likelihood that mutations in such sequences may affect transcription of both strands (Bogenhagen and Romanelli, 1988; L’Abbe et al., 1991; Randi and Lucchini, 1998).

The bi-genomic mito-nuclear genetic system presents four challenges: (A) whereas mtDNA-encoded proteins are already within the mitochondria, nDNA-encoded factors should be actively imported from the cytoplasm; (B) each cell harbors multiple mitochondria (∼1000 per human somatic cell), each requiring precise molecular numbers of nDNA-encoded factors; (C) each mitochondria harbors between 2 and 10 mtDNA molecules, which may differ in sequence thus creating a mixed population of mtDNAs per cell, tissues and organism, termed heteroplasmy (Larsson, 2010). In many cases heteroplasmic mutations have functional potential (Rebolledo-Jaramillo et al., 2014; Ye et al., 2014), which may in turn affect mito-nuclear interactions (see further discussion below). Finally, (D) in animals, the mtDNA evolves an order of magnitude faster than the nDNA (Brown et al., 1979; Castellana et al., 2011; Bar-Yaacov et al., 2012b). In this essay we will focus mostly on the fourth challenge, which mainly affects direct and epistatic interactions between mtDNA and nDNA-encoded factors. The solution to this challenge is facilitated by natural selection in a process termed co-evolution: functional mutations in the mtDNA could be compensated by additional functional changes, as has been claimed in the case of disease-causing mutations, either within the mtDNA or by co-evolution between genes encoded by the mtDNA and the nDNA. Since selection acts on phenotypes, the need for functional compensation is relevant only to mutations with phenotypic impact.

### mtDNA COMMON VARIANTS HAVE FUNCTIONAL IMPLICATIONS

The mtDNA was traditionally used by population geneticists as a neutral marker to track ancient population migration and to study the evolution of species. However, several lines of evidence show that mtDNA mutations have functional consequences. One of the first pieces of evidence came from a mutational screen for cell survival in the presence of chloramphenicol, an antibiotics agent that directly affects the mitochondrial protein translation machinery (Giles et al., 1980). This study, performed in cytoplasmic hybrids (cybrids) in mouse and human cells, revealed mutations in the mtDNA-encoded 16S ribosomal RNA that conferred resistance to chloramphenicol (Blanc et al., 1981). During the late 80s, the first disease-causing mutations were identified in the human mtDNA (Holt et al., 1988; Wallace et al., 1988a,b), which led to the subsequent discovery of many more mtDNA disease-causing mutations, thus fortifying the functional importance of mtDNA sequences. However, most disease-causing mutations cause devastating phenotypes, and hence are negatively selected and re-occur multiple times in unrelated families (Russell and Turnbull, 2014). Unlike disease-causing mutations, common genetic variants, of which most define nodes in the mtDNA phylogenetic tree, have survived selective pressures during the course of evolution, thus enabling their prevalence in the population. Although many such ancient (nodal) mtDNA genetic variants are neutral, some of them carry functional properties (Levin et al., 2013).

**FIGURE 2:**
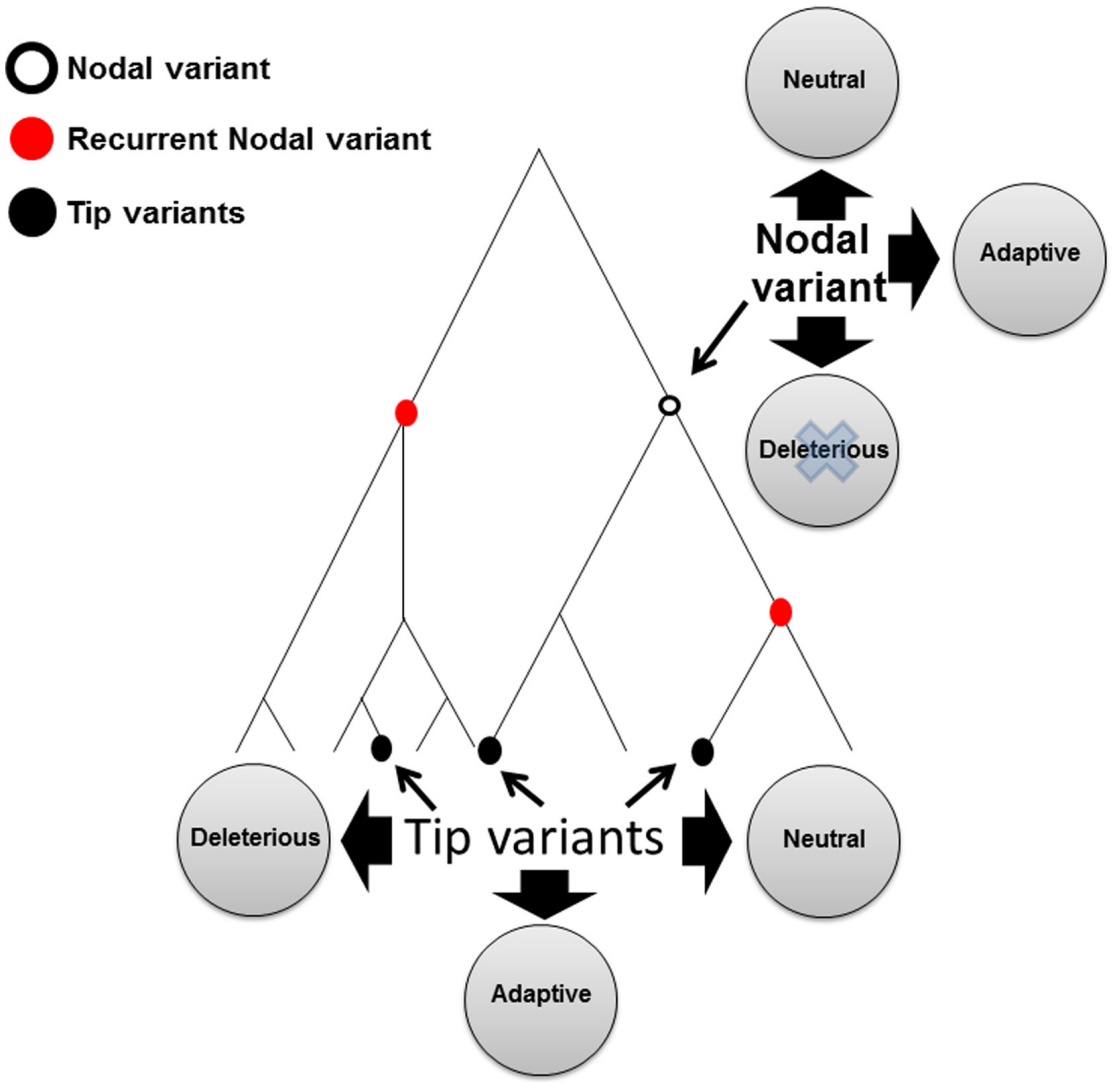
**A schematic phylogenetic tree illustrating tip, nodal, and recurrent nodal variants.** Tip variants, black filled circle; Nodal variant, black empty circle; RNM, red circle.

Many pieces of evidence support the functional implications of mtDNA common variants (Mishmar and Zhidkov, 2010). mtDNA common variants altered the penetrance of disease-causing mutations, such as that of Leber’s hereditary optic neuropathy (LHON; Brown et al., 2002; Howell et al., 2003; Hudson et al., 2007; Zhang et al., 2010). Association was discovered between mtDNA common variants and altered susceptibility to various types of complex diseases in humans such as type 2 diabetes (Mohlke et al., 2005; Fuku et al., 2007; Cormio et al., 2009; Feder et al., 2009), several heart diseases (Castro et al., 2006; Nishigaki et al., 2007; Kofler et al., 2009; Palacin et al., 2011; Strauss et al., 2013), a variety of neurological phenotypes (Chinnery et al., 2000; Carrieri et al., 2001; Pyle et al., 2005; Amar et al., 2007; Jones et al., 2007), age related macular degeneration (Heher and Johns, 1993; Jones et al., 2007; Canter et al., 2008; SanGiovanni et al., 2009; Udar et al., 2009; Mueller et al., 2012b; Kenney et al., 2013; Tilleul et al., 2013), but also of phenotypes such as longevity (Rose et al., 2001; Dato et al., 2004; Shlush et al., 2008; Cai et al., 2009; Dominguez-Garrido et al., 2009; Takasaki, 2009; Courtenay et al., 2012), and sperm motility (Ruiz-Pesini et al., 2000; Montiel-Sosa et al., 2006). Mammalian cell lines in which the mtDNA was depleted (Rho0 cells) and repopulated with different mitochondria carrying a diverged mtDNA genetic background showed differences in production of reactive oxygen species (Moreno-Loshuertos et al., 2006; Mueller et al., 2012a; Tiao et al., 2013), calcium uptake (Kazuno et al., 2006), OXPHOS function (Ji et al., 2012), and mtDNA copy number (Suissa et al., 2009). Hence, certain ancient common mtDNA variants have functional implications. Only in some cases it has been shown that functional mtDNA variants conferred adaptive advantage, as suggested in the case of human adaptation to different climates worldwide or high altitude in Tibetians ( Mishmar et al., 2003; Ruiz-Pesini et al., 2004; Ji et al., 2012). One could argue that in different from disease-causing mutations, functional common variants could either be mildly deleterious or confer adaptive properties, or otherwise they would have been removed due to negative selection (**Figure 2**). However alternatively, functional common mtDNA genetic variants could have survived a long evolutionary time because of functional compensation, or in its more common term – co-evolution.

### MITOCHONDRIAL AND nDNA-ENCODED FACTORS CO-EVOLVE AS A RESPONSE TO MUTATION RATE DIFFERENCES

The mutation rate difference between the mtDNA and nDNA could clearly lead to the occurrence of functional mtDNA mutations that interfere with mitochondrial activities relying on mito-nuclear epistasis and physical interactions (Gershoni et al., 2014). Such interactions include: (A) protein–protein interactions within OXPHOS protein complexes I, III-V (but not within OXPHOS complex II – succinate dehydrogenase – which comprises only nDNA-encoded proteins); (B) interactions between nDNA-encoded proteins and mtDNA-encoded RNA genes, i.e., rRNA and nDNA-encoded proteins within the mitochondrial ribosome and tRNAs with nDNA-encoded tRNA synthases; (C) interactions between nDNA-encoded proteins with mtDNA encoded mRNAs as part of post-transcriptional regulatory processes (Wolf and Mootha, 2014); and (D) interactions between nDNA-encoded transcription and replication factors (proteins and non-coding RNAs) with their mtDNA binding sites (Blumberg et al., 2014).

During the past decade it has been shown that natural selection has affected the mito-nuclear rate differences by tight co-evolution between interacting proteins encoded by the two genomes in the OXPHOS system (Yadava et al., 2002; Grossman et al., 2004; Rand et al., 2004; Schmidt et al., 2005; Meiklejohn et al., 2007; Rand, 2008). High-resolution 3D structures of OXPHOS complex IV have enabled investigation of co-evolution between nDNA and mtDNA-encoded subunits (Schmidt et al., 2001). Correlated mutations among mtDNA- and nDNA-encoded factors allowed us to predict and experimentally verify interactions between subunits of human OXPHOS complex I (Mishmar et al., 2006; Gershoni et al., 2010, 2014).

### MITO-NUCLEAR CO-EVOLUTION IN THE MITOCHONDRIAL PROTEIN TRANSLATION SYSTEM

The OXPHOS and the mitochondrial translation machineries are the only two mitochondrial machineries consisting of genes encoded both by the mtDNA and the nDNA. Similar to the OXPHOS system (which is discussed above), mtDNA-encoded rRNAs and their interacting nDNA-encoded ribosomal proteins (Smits et al., 2007; Desmond et al., 2011) have likely co-evolved to maintain structure and function of the ribosome (Barreto and Burton, 2013). Additionally, it has been reported that the RNA component of the mitochondrial ribosome is reduced in size compared to its bacterial homolog, with a compensatory increase in protein content to maintain the 3D structure (Mears et al., 2006; Brown et al., 2014; Greber et al., 2014a,b), further supporting mito-nuclear co-evolution. Co-evolution between the mtDNA-encoded rRNAs and their interacting proteins has yet to be studied, though accelerated mutation rate has been observed for the nDNA-encoded mitochondrial ribosomal proteins as compared to cytosolic ribosomal proteins (Barreto and Burton, 2013).

Investigating patterns of co-evolution among interacting factors within the mitochondrial ribosome requires high resolution structural information. Only recently, the structure of the mammalian mitochondrial large ribosomal subunit was resolved in a relatively high 3.4A resolution (Brown et al., 2014; Greber et al., 2014a,b; Kaushal et al., 2014). Such resolution enables deciphering the physical interaction between nDNA-encoded proteins and the mtDNA-encoded 16S rRNA; nevertheless, the interactions between proteins and the 12S rRNA within the small ribosomal subunit are yet to be defined. Since the closest structural relative of the mitochondrial ribosome is the bacterial one, and since the ribosomal tRNA entry point is highly conserved from bacteria to human (Ben-Shem et al., 2011), patterns of co-evolution within the bacterial ribosome may shed light on forms of evolutionary dynamics and interactions within the mitochondrial ribosome. Indeed, correlated changes have been observed between the 23S rRNA (the ortholog of mtDNA-encoded 16S rRNA) and a directly interacting protein – alpha helix 3 of ribosomal protein L11 in bacteria (Guha Thakurta and Draper, 1999). Such results emphasize the importance of understanding patterns of co-evolution within the mitochondrial ribosome as a proxy for physical interactions.

Another aspect of mito-nuclear RNA-protein co-evolution is reflected in the need for compatibility between the mtDNA-encoded tRNA Tyr and the nDNA-encoded tRNA Tyr-synthase to maintain normal development and mitochondrial function among *Drosophila* taxa (Hoekstra et al., 2013; Meiklejohn et al., 2013). The recent identification of mitochondrial mRNA-binding by proteins and miRNAs (Mercer et al., 2011; Liu et al., 2013; Wolf and Mootha, 2014; Zhang et al., 2014) may assist in isolating such binding factors and investigating their co-evolution with the bound mtDNA-encoded mRNAs. Such approaches may, in turn, assist in the identification of proteins involved in modes of mtDNA transcript modification, such as the recently discovered human mitochondrial RNA editing (Bar-Yaacov et al., 2013). In summary, mito-nuclear RNA-protein co-evolution is not restricted to the mitochondrial ribosome and could shed light on novel regulatory aspects of the organelle.

### MITOCHONDRIAL-NUCLEAR CO-EVOLUTION AND REGULATION

The genome of the mitochondrial ancestor is believed to have encompassed ALL the genes and information required for its actions. Similar to its free living relatives, contemporary mtDNA genes are jointly transcribed in a polycistrone, thus keeping their ancient prokaryotic mode of regulation. Accordingly, it is likely that genes within the genome of the mitochondrial free living bacterial ancestor were co-regulated as a polycistrone. However, the genes currently encoding mitochondrial activities, including the subunits of the OXPHOS protein complexes and the mitochondrial ribosome, are dispersed throughout the human genome and are mapped to different chromosomes apart from the mtDNA; hence, the problem of their co-regulation is a major issue as these factors have to collaborate within multi-subunit protein complexes in many different tissues. Indeed, co-expression has been identified among genes that encode protein subunits that participate in the same OXPHOS complexes (Duborjal et al., 2002; van Waveren and Moraes, 2008; Garbian et al., 2010). Accordingly, the expression pattern (mRNA) of genes belonging to the OXPHOS pathway was jointly altered in type 2 diabetes patients (Antonetti et al., 1995; Mootha et al., 2003). Micro-RNA based co-regulation of genes encoding protein subunits of the mitochondrial ribosome has been suggested (Ponsuksili et al., 2013). Furthermore, changes in the expression pattern of nDNA-encoded proteins have been described in cells grown from patients with mtDNA-encoded tRNA disease-causing mutations, suggesting signals delivered from the mitochondria to the nucleus (retrograde signaling) and coordinated regulation (Rabilloud et al., 2002; Chae et al., 2013; Picard et al., 2014). These pieces of evidence point to the possible existence of a mechanism (or mechanisms) that direct the co-regulation of mtDNA and nDNA-encoded factors of the OXPHOS, and possibly the mitochondrial translation, systems.

If such mechanism indeed exists, there should be factors that are involved in the joint regulation of mtDNA and nDNA genes. Indeed, some transcription factors (TFs), including NRF1, NRF2, PGC1a and YY1, have been identified as candidate regulators of proteins related to the OXPHOS system (van Waveren and Moraes, 2008; Leigh-Brown et al., 2010). NRF1 and NRF2 also modulate the transcription of mtDNA transcription regulators such as mitochondrial transcription factor A (TFAM; Scarpulla, 2008). Interestingly, two known regulators of mtDNA transcription, i.e., TFAM (Pastukh et al., 2007), and the mtDNA RNA Polymerase (POLRMT; Kravchenko et al., 2005), were localized and involved in transcription both in the mitochondria and in the nucleus, although nuclear functions of POLRMT have recently been questioned (Kuhl et al., 2014). Furthermore, tissue-specific methylation alterations of CpG dinucleotide have been observed in promoters of nDNA-encoded genes with mitochondrial function, implying tissue specificity in mitochondrial transcriptional regulation (Takasugi et al., 2010). Consistent with this finding, we have shown mitochondrial localization and direct human mtDNA binding of c-Jun and Jun-D in a cell-type specific manner (Blumberg et al., 2014). Similar to c-Jun and Jun-D, other TFs such as the thyroid hormone receptor, MEF2D and the glucocorticoid receptor, which are known regulators of the transcription of nDNA genes, are also imported into the mitochondria, where they bind the mtDNA and regulate its transcription ( Enriquez et al., 1999; Leigh-Brown et al., 2010; Psarra and Sekeris, 2011; She et al., 2011; Szczepanek et al., 2012; Blumberg et al., 2014). Finally, comparable to the components of mitochondrial transcription, some mtDNA replication components, such as hDNA2, APE1, Pif1, and DNA ligase III perform their activities both in the nucleus and in the mitochondria (Lakshmipathy and Campbell, 1999; Chattopadhyay et al., 2006; Futami et al., 2007; Duxin et al., 2009). Taken together, these discoveries indicate that nDNA and mtDNA transcription and replication could be co-regulated by a set of shared factors (Bar-Yaacov et al., 2012b).

The above findings imply co-evolution between factors that directly regulate mtDNA transcription and/or replication along with their mtDNA binding sites. This suggestion gained support by the finding that human POLRMT cannot bind and initiate transcription at the mouse light strand mtDNA promoter and vice versa (Gaspari et al., 2004). Additionally, human mtDNA genetic variants altered *in vitro* transcription and affected the binding capacity of TFAM (Suissa et al., 2009). Certain polymorphic variants in TFAM alter the susceptibility to develop Parkinson’s disease in Polish patients, in close correlation to the mtDNA genetic background haplogroup HV, suggesting that interfering with mito-nuclear interaction at the transcription level is involved in the etiology of the disease (Gaweda-Walerych et al., 2010). However, no such association was identified in the Spanish population (Alvarez et al., 2008), thus implying that other TFAM modulating factors are involved. Thorough investigation of the co-evolution between nDNA-encoded TFs and their mtDNA binding sites both within and between species should be performed in the near future. Such a study will be of special interest in light of the identified effect of male-specific mtDNA mutations in *Drosophila* on the expression of nDNA-encoded genes of the male reproductive system (Innocenti et al., 2011). As selective constraints are different in non-coding versus gene-coding sequences one should take into account the rapid change rate of non-coding regulatory elements within the mtDNA (Montooth et al., 2009). With this in mind, it is also plausible that there are regulatory elements that reside within the mtDNA-coding sequences, as was recently shown in the nDNA (Birnbaum et al., 2012). This suggests dual roles for such putative sites – they may code for genes but also promote binding of regulatory factors (Stergachis et al., 2013). Consistent with this suggestion we found that ChIP-seq binding sites of c-Jun, Jun-D and CEBPb in human mtDNA occur within protein coding genes and are negatively selected (Blumberg et al., 2014). A thorough screen for such mtDNA sites could pave the way toward new models of mito-nuclear co-evolution that take into account more than one type of selective constraints.

### THE INTRA-CELLULAR POPULATION GENETICS OF THE mtDNA AND ITS IMPLICATIONS ON MITOCHONDRIAL-NUCLEAR CO-EVOLUTION

Unlike the nDNA, the mtDNA resides in multiple cellular copies that may differ in sequence, thus creating an intra-cellular mixed population of heteroplasmic mtDNA molecules. This phenomenon adds another aspect to mito-nuclear interactions: intracellular diversity within a single cell and individual. Heteroplasmic mutations could have pre-existed at the mtDNA population within the ovum, but may also accumulate during the lifetime of the individual ( Goto et al., 2011; Avital et al., 2012). Notably, both inherited and accumulated heteroplasmy may vary in levels among cell-types due to unequal mitochondrial sorting after cell division. Additionally, levels of mtDNA inherited heteroplasmy could be modified due to mitochondrial bottleneck occurring at the pre-migratory germ cells during the development of the female embryo (Cree et al., 2008; Wai et al., 2008). Such bottleneck in combination with the varying energy demand of the different tissues might also play a role in explaining the variable penetrance of mtDNA mutations (Wolff et al., 2014): Deleterious mtDNA mutations and deletions will lead to a disease phenotype only when crossing a threshold of 80% (point mutations) or 60% (deletions) of the mtDNA population within tissues (Zeviani and Di Donato, 2004; Schapira, 2012). However, heteroplasmy patterns in identical (MZ) twins revealed that even low-prevalence heteroplasmic mutations are under strong negative selective constraints (Avital et al., 2012). Additionally, mtDNA disease-causing mutations were found in many normal individuals, though at very low levels of heteroplasmy (Rebolledo-Jaramillo et al., 2014; Ye et al., 2014). This suggests that heteroplasmic mutations at low levels of prevalence could have functional implications, thus implying for the active removal of dysfunctional mitochondria at the subcellular level by selective mechanisms such as mitophagy (Twig and Shirihai, 2011).

It is plausible that deleterious mutations, which are present at a heteroplasmic state, likely affect mito-nuclear interactions thus partially explaining the molecular basis underlying their phenotypic impact (Carelli et al., 2003). This is supported by the finding that mtDNA pathological mutations have partial penetrance which is modulated by nDNA modifiers (Carelli et al., 2003; Hudson et al., 2005; Shankar et al., 2008; Luo et al., 2013). Additionally, while artificially creating heteroplasmic cells with mixed mtDNA haplotypes, a skew toward over-representation of the mtDNA molecules from the same strain of the nDNA has been observed (Lee et al., 2008). These pieces of evidence suggest that mito-nuclear co-evolution is under strong selective constraints aimed to protect mito-nuclear interactions from mutations not only in the population, but also at the subcellular level.

### DISRUPTING MITO-NUCLEAR INTERACTIONS DRIVE EVOLUTION FORWARD

Interrupting with mito-nuclear co-evolution cause mitochondrial dysfunction, but not necessarily lead to disease phenotypes. Human cells in which the mitochondria were replaced either by chimpanzee or gorilla mitochondria (xenomitochondrial cybrid cells; Barrientos et al., 1998) exhibited a 40% reduction in the activity of OXPHOS complex I. Similar reduction in complex I activity was described in human children with inherited mutations in complex I subunits, which led to generalized hypotonia, developmental arrest and death before their second year of life (Rubio-Gozalbo et al., 2000). Hence, functional mito-nuclear incompatibilities of humans and our closest phylogenetic relatives affect mitochondrial function to a degree similar to known mitochondrial diseases. Likewise, reduced activity of OXPHOS complexes I and IV have been found in interspecific rodent cybrid cells (Dey et al., 2000; McKenzie and Trounce, 2000; Yamaoka et al., 2000). Moreover, backcross experiments have demonstrated that incompatibilities between the mitochondrial and nuclear genomes can affect mitochondrial function in *Drosophila* flies (Sackton et al., 2003) and mice (Roubertoux et al., 2003), sperm viability and morphology in the seed beetle (Dowling et al., 2007b), sex-specific fitness in *Drosophila* (Rand et al., 2001) and yeast (Lee et al., 2008), and mortality rate in the parasitoid wasps *Nasonia giraulti* and *Nasonia vitripennis* (Ellison et al., 2008; Niehuis et al., 2008). This implies that maintaining mito-nuclear genetic compatibility within species, but not between species, is a hallmark of evolutionary divergence.

Since maintenance of mito-nuclear co-evolution is importance for life, it is possible that its interruption in inter-population hybrids within the same species will associate with reduced fitness, thus marking insipient speciation events. A series of inter-population breeding experiments in the copepod *Tigriopus californicus* revealed reduction in hybrid fitness and activity of OXPHOS complexes (mainly complex IV; Burton et al., 2006; Ellison and Burton, 2010). Similarly, reduced fitness in inter-population breeding in *Drosophila* was attributed to mito-nuclear interactions (Rand et al., 2001; Dowling et al., 2007a). Cybrids carrying a single common human nDNA genetic background matched with a repertoire of mtDNAs from diverse lineages in human and mouse unveiled alterations in mitochondrial function (Kazuno et al., 2006; Moreno-Loshuertos et al., 2006; Kenney et al., 2014) and variation in nDNA gene expression (reviewed in Horan and Cooper, 2014). This likely occurred via signals carried by small molecules from the mitochondria to the nucleus, termed retrograde signaling (Kenney et al., 2014; also reviewed in Horan and Cooper, 2014). Finally, mito-nuclear incompatibility may have played a role in the divergence of natural sparrow populations (Trier et al., 2014) and possibly in other vertebrates (Bar-Yaacov et al., 2012a). Such observations have led to the suggestion that mito-nuclear incompatibility could play a role in the generation of reproductive barriers, an essential step toward the emergence of new species (Gershoni et al., 2009).

## INTERFERENCE WITH MITO-NUCLEAR INTERACTIONS LEADS TO ILLNESSES

Given that co-evolution between interacting factors is important to maintain function, it is reasonable that interfering with such co-evolution may alter phenotypes (Dowling, 2014). This thought led Theodosius Dobzhansky and Hermann Joseph Muller to independently suggest during the first half of the 20th century that alterations in one genetic element without compensatory response from its epistatic interacting partner could give rise to reproductive barriers and, eventually, speciation events (Dobzhansky, 1936; Muller, 1942; Gavrilets, 2003). Since interactions among mtDNA and nDNA-encoded factors are important for cellular function and since some modes of co-regulation exist, at least at the transcriptional level (Leigh-Brown et al., 2010), it is logical that disruption of the mito-nuclear association may cause diseases. Indeed, the penetrance of mtDNA mutations that cause LHON was shown to be modulated by X-linked nDNA encoded elements (Hudson et al., 2005; Shankar et al., 2008). nDNA-encoded modifying factors of mtDNA mutations that underlie hearing loss were also suggested (Johnson et al., 2001; Guan, 2011; Kokotas et al., 2011; Luo et al., 2013). A combination of mtDNA and nDNA modifying factors for Huntington disease was recently proposed (Taherzadeh-Fard et al., 2011). Interaction between nDNA and mtDNA genotypes was shown to affect male fertility in *Drosophila* (Yee et al., 2013). A recent repeated backcross experiment study which led to mitochondrial nuclear exchange mice showed, that mito-nuclear genetic interactions alter the susceptibility to non-alcoholic fatty liver disease (NAFLD; Betancourt et al., 2014). Similarly, conplastic strains of rats, i.e., strains sharing their nDNA yet diverge in their mtDNA sequences including amino acid mutations in OXPHOS subunits, show altered risk to develop type 2 diabetes (Pravenec et al., 2007) and differential cardiac functions (Kumarasamy et al., 2013). Accordingly, the association of certain mtDNA genetic backgrounds with a tendency to develop type 2 diabetes mellitus is modified by nDNA-encoded genes in Jews (Feder et al., 2009; Gershoni et al., 2014), Italian (Cormio et al., 2009), and in Indian patients (Rai et al., 2007). The disease-causing phenotype of a mutation in *NDUFA1*, an nDNA-encoded subunit of OXPHOS complex I, is partially modulated by mtDNA-encoded factors (Potluri et al., 2009). Strauss et al. (2013) found differences in the severity of cardiomyopathy among homozygous patients to the frame-shift mutation (p.Q175RfsX38) in the nDNA-encoded *SCL25A4* gene depending whether they also carried the mtDNA haplogroup U in comparison to patients with mtDNA haplogroup H. Taken together, all these findings suggest that disruption of the connection between certain mtDNA-nDNA-encoded factors can lead to diseases.

## BEYOND MITO-NUCLEAR CO-EVOLUTION: THE SURVIVAL OF mtDNA FUNCTIONAL NODAL MUTATIONS THROUGH ADAPTATION, COMPENSATION, AND FUNCTIONAL CONVERGENCE

**FIGURE 3:**
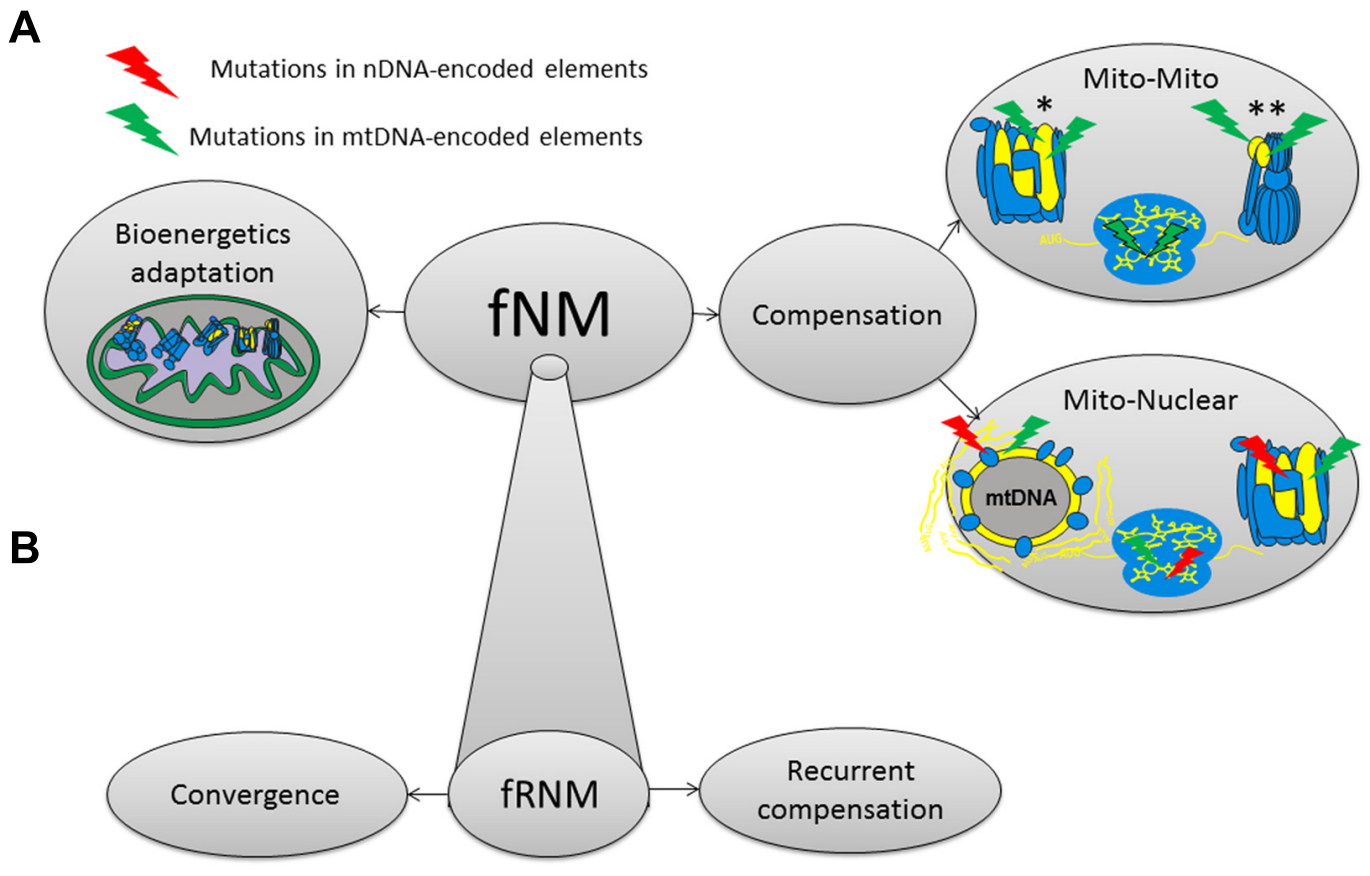
**A model summarizing mechanisms underlying the long term evolutionary survival of functional nodal mutations (fNMs). (A)** fNMs, functional nodal mutations. *The compensatory mutation and the fNM occurred within the same mtDNA encoded component. **The compensatory mutation and the fNM occurred on different mtDNA encoded components. **(B)** Possible mechanisms underlying the long term evolutionary survival of functional recurrent nodal mutations (fRNMs).

Until now we underlined the co-evolution between mtDNA and nDNA-encoded elements as a mechanism that responds to the order of magnitude difference in mtDNA and nDNA mutation rates, thus enabling maintenance of mitochondrial function. However, the long term evolutionary survival of mtDNA functional mutations could be enabled by mechanisms other than compensatory nDNA mutations (**Figure 3**). It is also possible that functional mtDNA mutations will be compensated by other mtDNA changes either within the same gene or elsewhere in the same genome (Kern and Kondrashov, 2004; de Magalhaes, 2005; Azevedo et al., 2006, 2009). Notably, compensations via newly arising mutations are more apparent while considering deep phylogeny which allows sufficient time for the accumulation of mutations, but in the frame of population divergence one could envision compensation that stem from pre-existing mutations, either within mtDNA genetic backgrounds or in the nDNA. Alternatively, functional mtDNA mutations could carry adaptive values which will support their long term survival because of positive selection, as was already shown for some ancient mtDNA functional variants (Mishmar et al., 2003; Ruiz-Pesini et al., 2004; Gu et al., 2012; **Figure 3**). Therefore, all three mechanisms, namely compensation via mito-nuclear co-evolution, compensation via mito-mito co-evolution and positive selection are plausible and should be considered as explanations for the survival of functional ancient variants in the mtDNA.

The need for mechanisms allowing the survival of functional ancient mutations is not restricted to mutations that appeared and survived only once during evolution, thus defining certain phylogenetic branches [i.e., single nodal mutations (SNMs)]. It is further emphasized, yet more complicated, in the case of functional nodal mutations (fNMs) that re-occurred during mtDNA phylogeny, i.e., recurrent nodal mutations (RNMs; **Figure 2**). Whereas the functional solutions for SNMs could be different for each of the various SNMs that define the various mtDNA phylogenetic branches, one could envision the emergence of similar and even identical mechanisms that recurrently allowed the survival of functional RNMs (Levin et al., 2013). This raises the intriguing possibility that functional RNMs may teach us about possible convergence in the mechanisms underlying functional compensation. Specifically, functional convergence implies that either similar compensatory solution, or similar adaptive value, underlies the survival advantage of functional RNMs defining distant branches in the human mtDNA phylogeny. It will therefore be of interest to assess whether functional RNMs played an adaptive role to similar traits in the phylogenetic branches in which they occurred. This option is currently investigated in our laboratory. Although the high mtDNA mutation rate makes the mtDNA the perfect model to study functional convergence, this phenomenon could explain the survival of functional SNMs and RNMs in other, non-mitochondrial, systems, thus offering general implications for functional convergence of ancient mutations.

## Conflict of Interest Statement

The authors declare that the research was conducted in the absence of any commercial or financial relationships that could be construed as a potential conflict of interest.
